# Microscopic and Molecular Detection of Camel Piroplasmosis in Gadarif State, Sudan

**DOI:** 10.1155/2017/9345231

**Published:** 2017-02-15

**Authors:** Abdalla Mohamed Ibrahim, Ahmed A. H. Kadle, Hamisi Said Nyingilili

**Affiliations:** ^1^Abrar Research and Training Centre, Abrar University, Mogadishu, Somalia; ^2^College of Veterinary Medicine, University of Bahri, Khartoum, Sudan; ^3^ICRC, Mogadishu, Somalia; ^4^Vector and Vector Borne Diseases Institute, Tanga, Tanzania

## Abstract

The socioeconomic importance of camels (*Camelus dromedarius*) could not be neglected in the Sudan. The present study was planned to confirm the presence of piroplasmosis in camels from the Eastern region of the Sudan (Gedarif State) using microscopical (blood film) and molecular technique (PCR). A total of 55 camels of different sexes (34 females and 21 males) were sampled from four localities of the state between January 2011 and January 2012. The prevalence rates using parasitological and molecular examinations were 43.6% and 74.5%, respectively. The prevalence rates significantly vary between the localities (*p* = 0.011) but not between the different sexes (*p* = 0.515). PCR technique showed higher sensitivity than microscopy. The present paper was to be the first report investigating camel piroplasmosis using both parasitological and molecular methods in the Eastern region of the Sudan. Further studies in the phylogenetic sequencing are to be continued for parasite speciation. Moreover, studies on the clinical and economic consequences of camel piroplasmosis are recommended.

## 1. Introduction

Sudan has the second largest camel population in the world, estimated at nearly 4,000,000, and owns 17% of the total world camel population [1]. Camels in the Sudan are receiving more attention, as they constitute a major component of livestock export to the neighboring countries. The camel “district” zone in the Sudan runs from the Eastern frontiers where camels come in contact with Ethiopian and Eritrean camel's herds to the Western frontiers where they can mix with the Chadian herds.

Causing serious economic losses tick and tick borne diseases (T and TBDs) still remain to be a major threat to animal's industry in the Sudan [2, 3] (El hussein et al., 2004; Hassan, and Salih 2009). The role of biting flies in the epidemiology of animal piroplasmosis was also discussed in the Sudan [4]. The most prevalent tick species affecting camels in the Sudan is* Hyalomma dromedary* in addition to other* Hyalomma* sp.,* Amblyomma* sp., and* Rhipicephalus* sp. (Hassan, and Salih 2009).

Very few camel piroplasmosis reports are available recently in the one-humped camel zone, such as Egypt [5], Iraq [6], and Iran [7]. With the exception of Abdelrahim et al. [8], there is not any report available on camel's TBDs from the Sudan.

*Babesia caballi* was molecularly detected from Sudanese camel [8] using Reverse Line Block** (**RLB). Both* Babesia caballi *and* Theileria equi* were molecularly confirmed in camels from Iraq [6] using PCR. Therefore, equines are supposed to play an important role in the epidemiology of camel piroplasmosis because they are usually found to be infected with the same piroplasms species [9, 10].

Clinical, haematological, and biochemical changes induced by naturally occurring babesiosis in dromedary camels were described by [11] in Kingdom of Saudi Arabia (KSA).

Among the few common diseases affecting camels, camel TBDs are usually neglected in the Sudan (Hassan, and Salih 2009) [12]. Many old and unpublished reports stated that camels are not susceptible to TBDs, although Shommein and Osman [13] earlier suspected that theileriosis, ehrlichiosis, and babesiosis may also be responsible for morbidity and mortality rates in camels. The economic impact of tick and tick borne diseases (T and TBDs) has inspired researchers to investigate TBDs in many animal species. However, in Sudan in spite of having the second largest counts of camels, data on camel piroplasmosis is not available. The present study was designed to determine the presence of piroplasms in one-humped camel in the Eastern region of the country using parasitological (microscopic) and molecular (PCR) techniques.

## 2. Materials and Methods

### 2.1. Study Area

**Figure 1:**
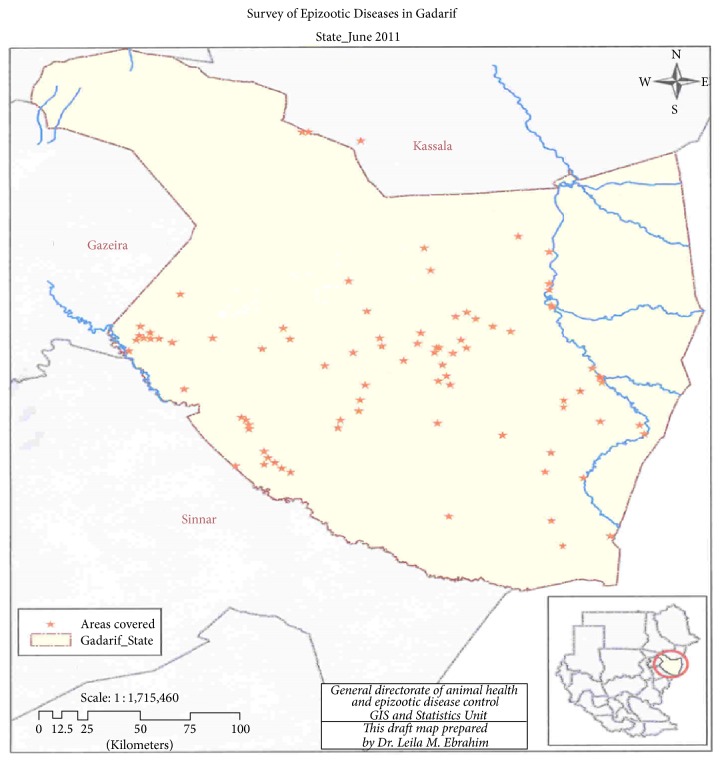
The sampled site in the study area (Gadarif State).

Al Gadarif is one of the 18 states of the Sudan and one of the three states of Eastern region of the country. It shares an international border with Ethiopia to the East. The state shares borders with four Sudanese states including, Kassala and Khartoum States to the north, Al-Jazira State to the west, and Sennar to the south (Figure 1). It is located between longitudes 33° 30 and 36° 30 East, and latitudes 12° 40 and 15° 46 North. It has an area of 75,263 km^2^ and an estimated population of approximately 1,400,000 (2000). It is one of the best agricultural (farms and livestock) areas in the Sudan. Four out of the ten localities of the state were included in this study, namely, Gadarif, Butana, Rahad, and Gala'alnahal.

### 2.2. Animals

Dromedary camels (*Camelus dromedarius*) were sampled among other animal's species for blood parasite investigation including piroplasms. These camels were sampled during area wide project entitled Survey for Epizootic Diseases. The project was designed by Ministry of Livestock, Fisheries and Range Lands (MLFRL), Sudan, in the year 2011. A total of 55 apparently healthy camels (34 females and 21 males) were included in this study.

### 2.3. Blood Samples

Fifty-five heparinized camel's blood samples (34 females and 21 males) were collected from jugular vein. Thin dried fixed blood smears and blood spot on Whatman No. 4 filter paper were prepared at the sample site. These samples were transported to Khartoum and submitted for further laboratory work in Laboratory of Parasitology, College of Veterinary Medicine, Sudan University of Science and Technology (SUST), Sudan. The thin dried fixed blood smears were stained using Giemsa's protocol and examined microscopically for presence of any blood parasites including piroplasms. The dried blood spots on filter paper were stored in −20°C until shipped to Vector and Vector Borne Diseases Institute (VVBDI), Tanga, Tanzania. These samples were investigated molecularly for presence of camel piroplasmosis in VVBDI using Polymerase Chain Reaction (PCR).

### 2.4. Extraction of the DNA

More than 10 micropunches of 1.2 mm each were taken from the preserved dry spot of whole blood on the filter paper. To reduce the chances of missing out piroplasm DNA punches were done randomly and kept on sterile 1.5 *μ*L Eppendorf tube. The well cleaned Harris 1.2 mm micropunch (Whatman Biosciences Ltd.) was used. To prevent contamination between samples, the punches were cleaned after every sample using a 70% ethanol; then punches were used to cut a clean filter paper before using it on the next sample.

Total DNA was isolated by fastest version of the chelex extraction technique. Briefly 200 *μ*L volume of solution with Chelex 100 (Sigma-Aldrich, St. Louis, USA) (final concentration 20%) was added to the 1.5 *μ*L Eppendorf tube with samples boiled for 10 min and preserved in −20°C and were spanned at 13000 rpm for 3 minutes before using 2 *μ*L of supernatant for PCR.

### 2.5. Polymerase Chain Reaction (PCR)

Extracted DNA samples were subjected to Internal Transcribed Spacers (ITS1) Polymerase Chain Reaction (PCR) amplification. Presence of piroplasms was characterized by PCR using the primers Bab-sp-F (GTTTCTGCCCCATCAGCTTGAC) and Bab-sp-R (CAAGACAAAAGTCTGCTTGAAAC) which were used as the forward and reverse primers, respectively [7, 14]. Both primers were supplied by Bioneer Corporation. The PCR amplifications were performed in a total reaction volume of 25 *μ*L containing 0.5 *μ*L of 10 pM of each primer, 12.5 *μ*L of one 2x master mix (BioLab. new England), 9.5 *μ*L of PCR water, and 2 *μ*L of each DNA template. PCR amplifications were performed with a thermal cycler (Gene Amp 9700 PCR system, Applied Biosystems). Amplification condition was initial denaturation at 94°C for 1 min and 30 seconds, followed by 45 cycles of 94°C for 20 s, 65°C for 30 s, followed by 68°C for 30 min, and final extension at 68°C for 10 min.

To ensure that results were not biased by false positives during repeated PCRs, negative controls in which DNA templates were replaced with sterile water as well as positive control DNA were included in all PCR reactions. The amplified PCR product was electrophoresed on a 1.5% agarose gel in 1x TBE. Quick loading 100 bp DNA ladder (BioLab, New England) was included on each gel, stained by ethidium bromide, run at 100–120 V for 60 min, and final visualized in Uvidock (Cambridge, UK).

The DNA template of a clear positive sample microscopically showing the characteristic pyriform and single amoebic form of* Babesia* sp. was firstly extracted and checked repeatedly by PCR to be used as positive control.

### 2.6. Data Management and Analysis

Data were stored in a Microsoft® Excel spread sheet for Windows® 2007 before being transferred to SPSS sheet for Windows version 20. The differences were considered statistically significant when *p* ≤ 0.05.

Photos of the detected parasites were captured directly from microscope eye piece using digital camera (Sony, 16.1 MP) and stored in computer.

## 3. Results

### 3.1. Microscopic Prevalence of Camel Piroplasmosis

**Figure 2:**
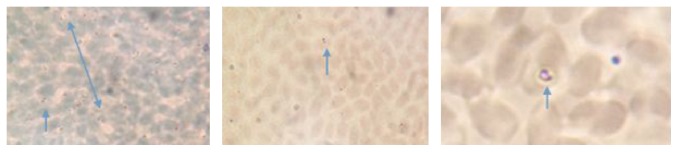
Piroplasms with different shapes (arrows) in Giemsa's stained blood.

Piroplasms were detected microscopically in Giemsa's stained blood films of 43.6% of the examined camels. The different shapes of detected parasites were presented in Figure 2 (arrows). Rahad locality revealed the highest microscopical prevalence rate (68.2%) with highly statistically significant (*p* = 0.011) variation from Gadarif (42.9%) and Gala'alnahal (28.6%) localities (Table 1). No piroplasm (0.0%) was detected microscopically in Butana locality.

### 3.2. Molecular Prevalence of Camel Piroplasmosis

**Figure 3:**
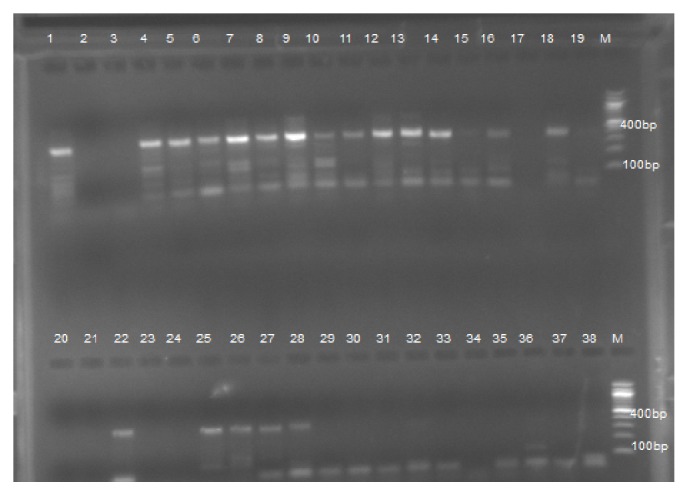
Agarose gel (1.5%) electrophoresis of amplified DNA from* Babesia. *Lane M: DNA ladder (100 bp). Lane 1: positive control and Lane 20: negative control. Positive product showed clear band in 400 bp (e.g., Lanes 4, 5, 6, 7, 8, 9, 12, 13, 14, 18, 22, 25).

Babesia DNA was detected molecularly in the extracted blood of 74.5% of the examined camels. The bands of the positive and negative Babesia DNA product were presented in Figure 3. Without any statistical significance (*p* = 0.328), Gadarif locality revealed the highest molecular prevalence rate (100%) followed by Gala'alnahal (76.2%) and Rahad (68.2%) localities (Table 1). Piroplasm DNA with prevalence rate of 60% was detected molecularly in Butana locality.

### 3.3. Prevalence of Camel Piroplasmosis in Different Sexes

Using both microscopical or molecular examination, there was not any statistically significant (*p* = 0.515 or 0.391) variation between male and female camels in the susceptibility of piroplasms infection. However, when male revealed higher susceptibility molecularly, female showed higher prevalence rate microscopically (Table 2).

### 3.4. Level of Agreement between Microscopical and Molecular Tests

PCR technique detected more infection (74.5%) than microscopical one (43.6%). PCR technique detected 22 (71.0%) and 19 (79.2%) out of the 31 negative and 24 positive samples microscopically, respectively (Table 3). The level of agreement between the two techniques is very poor (Kappa = 0.076).

## 4. Discussion

The socioeconomic value of Sudanese camels is well recognized nationally, regionally, and internationally. The most important pathogenic and epidemic diseases affecting camels in the Sudan are of parasitic origin. There are very few and sporadic serious diseases of viral and bacterial origin. With the exception of the single case of molecular camel babesiosis [8], there is not any report on camel's TBDs available from the Sudan.

The present study revealed that more than two-thirds (74.5%) of camels of the investigated area were found to be infected with piroplasmosis. The prevalence of camel piroplasmosis using microscopical examination in this study was higher than that reported in KSA [11], Egypt [5], and Iraq [6]. This could be attributed to the higher prevalence rate of ticks and biting flies infesting camel in the investigated area of the Sudan [2] (Hassan, and Salih 2009). This was clearly confirmed when the molecular results of the present study were found to be also higher than that recorded by Khamesipour et al. [7] in Iran and Jasim et al. [6] in Iraq. Only one camel (0.5%) sample out of 200 samples from Western region of the Sudan showed* Babesia caballi* DNA [8] using the Reverse Line Block (RLB). It is incomparable with the present results and that is may be only due to the different molecular technique (ITS1-PCR) used in this study.

The results of this study revealed that PCR is more sensitive for detection of camel piroplasmosis than microscopy. Similar observations were reported in camels from Iran [7] and Iraq [6].

In this study, about 75% of the investigated camels were found to be positive for piroplasmosis. However, no pathognomonic clinical signs of piroplasmosis (e.g., haemoglobin-urea) were recorded during sampling. Swelum et al. [11] reported some clinical, haematological, and biochemical changes due to natural infection of babesiosis in dromedary camels in Kingdom of Saudi Arabia (KSA). Camel is known to be tolerant to many diseases. However, from the present results, the effect of piroplasmosis in degree of anaemia as well as production and the productivity of camel need to be investigated in depth. Additionally, when mix-infection of protozoan parasites causing immunosuppression is present, the impact of the disease will be higher [3, 15]. Based on the present results, camel could be a source of infection for the coherded equines and vice versa, because camels are found to be infected by equine piroplasms including* Babesia caballi *and* Theileria equi* [6, 8]. Thus, camels should be considered in the epidemiology of equine piroplasmosis [9, 10].

From the results of the present study, female camels showed more acute infection when more parasitaemia was detected microscopically than males, although sex has no significant effect (*p* > 0.05) in the susceptibility of infection.

Ticks are widespread in camel-raising habitats in the Sudan [2] (El hussein et al., 2004; Hassan, and Salih 2009). They cause serious adverse effects such as anaemia, dermatitis, mastitis, reduced meat and milk production, and low quality hides. The high prevalence of piroplasmosis revealed in this study could explain that TBDs may seriously affect the production and the productivity of camels in the Sudan. Moreover, the present results come to support the earlier suggestion of Shommein and Osman [13] that TBDs could be responsible for morbidity and mortality rate of Sudanese camels. Therefore, we come to conclude that to improve camel production and productivity in the Sudan, it is high time to monitor camels from tick borne diseases and to implement prophylaxis and treatment. Further study in the phylogenetic sequence of these DNA templates is recommended for parasite speciation.

## Figures and Tables

**Table 1:** Prevalence of camel piroplasmosis in the investigated localities.

Locality	*N*	Prevalence *n* (%)	
		Parasitological	PCR
Butana	5	0 (0.0)	3 (60)
Gadarif	7	3 (42.9)	7 (100)
Gala'anahal	21	6 (28.6)	16 (76.2)
Rahad	22	15 (68.2)	15 (68.2)

Total	55	24 (43.6)	41 (74.5)

*p value*		*0.011*	*0.328*

**Table 2:** Prevalence of camel piroplasmosis in different sexes.

Sex	*N*	Prevalence *n* (%)	
		Parasitological	PCR
Male	21	8 (38.1)	17 (81.0)
Female	34	16 (47.1)	24 (70.6)

Total	55	24 (43.6)	41 (74.5)

*p* value		0.515	0.391

**Table 3:** The level of agreement between the two techniques using Kappa test.

		Molecular (PCR)		Total
		N−ve	P+ve	
Microscopic (BF)	N−ve	9 (29.0)	22 (71.0)	**31 (100)**
	P+ve	5 (20.8)	19 (79.2)	**24 (100)**

Total		14 (25.5)	41 (74.5)	**55 (100)**

Kappa value		**0.076**		
